# Cartilage protective and anti-analgesic effects of ALM16 on monosodium iodoacetate induced osteoarthritis in rats

**DOI:** 10.1186/s12906-019-2746-7

**Published:** 2019-11-21

**Authors:** Doo Jin Choi, Soo-Im Choi, Bo-Ram Choi, Young-Seob Lee, Dae Young Lee, Geum Soog Kim

**Affiliations:** 1 Department of Herbal Crop Research, National Institute of Horticultural and Herbal Science, RDA, Eumseong, 27709 Republic of Korea; 2 Plant Resources Research Institute, Duksung Women’s University, Seoul, 01369 Republic of Korea

**Keywords:** Osteoarthritis (OA), *Astragalus membranaceus*, *Lithospermum erythrorhizon*, Matrix metalloproteinases (MMPs), Monosodium iodoacetate (MIA)

## Abstract

**Background:**

Osteoarthritis (OA) is an age-related joint disease with characteristics that involve the progressive degradation of articular cartilage and resulting chronic pain. Previously, we reported that *Astragalus membranaceus* and *Lithospermum erythrorhizon* showed significant anti-inflammatory and anti-osteoarthritis activities. The objective of this study was to examine the protective effects of ALM16, a new herbal mixture (7:3) of ethanol extracts of *A. membranaceus* and *L. erythrorhizon,* against OA in in vitro and in vivo models.

**Methods:**

The levels of matrix metalloproteinase (MMP)-1, −3 and − 13 and glycosaminoglycan (GAG) in interleukin (IL)-1β or ALM16 treated SW1353 cells were determined using an enzyme-linked immunosorbent and quantitative kit, respectively. In vivo, the anti-analgesic and anti-inflammatory activities of ALM16 were assessed via the acetic acid-induced writhing response and in a carrageenan-induced paw edema model in ICR mice, respectively. In addition, the chondroprotective effects of ALM16 were analyzed using a single-intra-articular injection of monosodium iodoacetate (MIA) in the right knee joint of Wister/ST rat. All samples were orally administered daily for 2 weeks starting 1 week after the MIA injection. The paw withdrawal threshold (PWT) in MIA-injected rats was measured by the von Frey test using the up-down method. Histopathological changes of the cartilage in OA rats were analyzed by hematoxylin and eosin (H&E) staining.

**Results:**

ALM16 remarkably reduced the GAG degradation and MMP levels in IL-1β treated SW1353 cells. ALM16 markedly decreased the thickness of the paw edema and writhing response in a dose-dependent manner in mice. In the MIA-induced OA rat model, ALM16 significantly reduced the PWT compared to the control group. In particular, from histological observations, ALM16 showed clear improvement of OA lesions, such as the loss of necrotic chondrocytes and cartilage erosion of more than 200 mg/kg b.w., comparable to or better than a positive drug control (JOINS™^,^ 200 mg/kg) in the cartilage of MIA-OA rats.

**Conclusions:**

Our results demonstrate that ALM16 has a strong chondroprotective effect against the OA model in vitro and in vivo, likely attributed to its anti-inflammatory activity and inhibition of MMP production.

## Background

Osteoarthritis (OA) is a degenerative disease characterized by progressive loss of articular cartilage in local joints and secondary changes and pain symptoms, especially in the aging population. Therefore, elderly people are increasingly interested in healthy foods and drugs that can be used to prevent or treat OA as the aging society is increasing around the world. Several previous studies have reported that major pathophysiologic features of OA are biochemical changes of cartilage and synovial membrane, formation of osteophytes, abrasions of articular cartilage, and subchondral bone sclerosis [1, 2]. The cause of OA is associated with the disruption of cartilage homeostasis, which is a physiological imbalance of synthesis and degradation in articular cartilage [3]. The articular cartilage destruction in OA, which leads to chronic pain and functional loss in the joints, is known to be induced by diverse factors such as excessive synthesis of inflammatory cytokines and activation of mechanical factors [4]. Among these factors, the action of degradative enzymes of all mechanistic classes causes the loss of extracellular matrix (ECM) components, including proteoglycans and collagen, in articular cartilage [5]. Previous studies have reported that matrix metalloproteinases (MMPs) produced by chondrocytes in response to inflammatory factors, including interleukin (IL)-1β, IL-6, and tumor necrosis factor (TNF)-α, play crucial roles in both tissues remodeling and the development of articular cartilage destruction in OA [6]. MMPs are a family of proteinases that degrade ECM proteins, including glycoproteins and collagens, and are classified into five main groups according to their function, structure and localization: collagenases (MMP-1, − 8, and − 13), stromelysins (MMP-3, − 7, − 10, and − 11), gelatinases (MMP-2 and -9), matrilysins and membrane type (MT)-MMPs [7]. Several studies have reported that inhibition of the proteolytic activity and expression of specific MMPs seems to block the progression of articular cartilage destruction [8, 9].

*Astragalus membranaceus*, known as hwangki in Korea, is one of the most widely used traditional medicinal herbs in Asian countries. It has been well established that *A. membranaceus* is enriched with triterpene saponins, flavonoids and polysaccharides [10]. Currently, more than 200 compounds have been identified from *A. membranaceus*, and these compounds have been reported to possess a variety of biological activities such as immunomodulating, anti-hyperglycemic, anti-tumor, and anti-neurodegenerative effects [11–14].

*Lithospermum erythrorhizon* has been used to treat various symptoms in Asian countries. *L. erythrorhizon* mainly contains naphthoquinone pigments, including shikonin and its derivatives [15]. Extracts of *L. erythrorhizon* have been reported to have osteogenic activity by modulating osteoblast differentiation, anti-oxidant, anti-inflammatory and anti-cancer effects [16–19].

In our previous study, we reported that ethanol extracts of *A. membranaceus* and an isoflavonoid, calycosin-7-*O*-*β*-D-glucopyranoside (CG) treatment significantly inhibited the matrix degradation caused by recombinant human IL-1β or hyaluronidase in human articular cartilage explants and chondrocytes [20]. In addition, injection of CG injection into cartilage significantly alleviated the OA-induced accumulation of prostaglandin (PG) and total proteins in synovial fluid and reduced the severity of the structural damage in cartilage caused by the pathogenesis of OA-like lesions in a rabbit model [21]. Shikonin and acetylshikonin isolated from *L. erythrorhizon* have also been reported to exhibit chondroprotective effects via inhibition of MMP production [22]. In spite of the health benefits of the two abovementioned herbs, the synergistic effects of a mixture of extracts from them have not been studied. Supplementary, we confirmed that ALM16, which were mixed at an optimal ratio, were found to have a higher inhibitory effect on MMPs levels induced by IL-1β than the individual source. Thus, this study was designed to clarify the potential applications of two herbal mixtures based on its synergistic activity, and to evaluate its nociceptive and protective effects the pathogenesis of articular cartilage in an OA model in vitro and in vivo. In present study, ALM16 was evaluated the chondroprotective and synergistic effects on IL-1β treated SW1353 cells by measuring the levels of MMPs and GAGs, and then was further confirmed the anti-osteoarthritis and analgesic effects using OA animal models.

## Methods

### Materials and reagent

Human SW1353 chondrosarcoma cells (ATCC® HTB-94™) were purchased from the American Type Culture Collection (Rockville, MD, USA). Dulbecco’s modified Eagle’s medium (DMEM), fetal bovine serum (FBS), penicillin and streptomycin and phosphate buffer saline (PBS) were purchased from GIBCO-BRL (Grand Island, NY, USA). Monosodium iodoacetate (MIA), 3-[4,5-dimethylthiazol-2-yl]-2,5-diphenyltetrazolium bromide (MTT), λ-carrageenan and acetic acid were purchased from Sigma-Aldrich (St Louis, MO, USA). The standard compounds, calycosin, calycosin-7-*O-β*-D-glucoside and lithospermic acid, were purchased from the Chem Faces (Hubei, China).

### Experimental animals

Male ICR mice (18–22 g, 6 weeks old) and Wistar/ST rats (120–140 g, 6 weeks old) were obtained from Orient Bio Co., Ltd. (Seongnam, Korea). ICR mice were used to evaluate acetic acid-induced writhing response and establish the carrageenan-induced paw edema model. Wistar rats were used for the MIA-induced OA model. All animals were maintained in a controlled room at 22 ± 0.5 °C and 55 ± 5% humidity with a light/dark period of 12 h for at least 1 week before used. The procedures used in this study were in agreement with the NIH Guidelines for the Care and Use of Laboratory Animals. The experimental protocol was made efforts to minimize the number of animals used in experiment and approved by the Animal Research Ethics Committee (YD Life Science. Co., Ltd.).

### Preparation of ALM16

*A. membranaceus* and *L. erythrorhizon* were cultivated in Jecheon (Chungcheongbuk-do, Korea). An *A. membranaceus* voucher specimen (MPS005087) and *L. erythrorhizon* voucher specimen (MPS004961) were taxonomically identified by Ph.D. Jeong Hoon Lee (National Institute of Horticultural and Herbal Science, Rural Development Administration). Dried roots of *A. membranaceus* and *L. erythrorhizon* were extracted by a heat reflux method at 80 °C for 4 h with 50 and 70% aqueous fermented ethanol, respectively, which was repeated 2 times. Each filtrate was concentrated in vacuum at 60 °C or lower to obtain 50 ± 1 and 66 ± 1 brix materials, respectively. These extracts were sterilized at 80–90 °C for 1 h and dried under reduced pressure (− 0.08 MPa) at 60–70 °C, and then, each dried solid was pulverized to obtain their extract powders. Each extract powder was mixed together at a ratio of 7:3 (w/w) to prepare the final extract mixture (ALM16). The powdered samples were stored at − 20 °C and used for in vitro experiments after being dissolved in DMSO.

### High-performance liquid chromatography (HPLC) analysis

The contents of calycosin and calycosin-7-*O*-*β*-D-glucoside from *A. membranaceus* extract (A extract) and lithospermic acid from *L. erythrorhizon* extract (L extract) were measured by HPLC-DAD as active compounds of each plant sample. For HPLC analysis, a YMC ODS-AM (4.6 × 250 mm, 5 μm) column was used at 30 °C with 0.1% formic acid and acetonitrile as the gradient system of the mobile phase in Water e2695 series HPLC system (Waters Corporation, Milford, MA, USA). The mobile phase was maintained at 5% acetonitrile for 3 min, increased to 20% for 3 min and then increased to 28% for 25 min. Then, elution was performed by increasing the mobile phase to 100% acetonitrile for 4 min. The flow rate was 1 ml/min, and the absorbance of the UV detector was measured at a wavelength of 254 nm.

### SW1353 chondrocyte cell culture and alginate beads

SW1353 cells were cultured in complete medium containing DMEM, 100 units/ml penicillin, 100 μg/ml streptomycin, and 10% FBS and were incubated in 5% CO_2_ at 37 °C. Chondrocytes were prepared for culture in alginate beads [23]. SW1353 cells were re-suspended at a density of 4 × 10^6^ cells/ml in a 1.2% (w/v) solution of sterile sodium alginate in 0.15 M NaCl. The cell suspension was slowly expressed with 22-gauge needle and dropped into the gel-forming solution (102 mM CaCl_2_). Beads with approximately 1 × 10^4^ cells/bead (3 mm in diameter) were allowed to polymerize for 10 min and washed twice with 0.15 M NaCl. The hydrogel beads were then transferred to DMEM (20 beads/well) in a 12-well plate containing 10% FBS. The beads were cultured with 5% CO_2_ at 37 °C for 2 weeks. The culture medium was refreshed every 2 days.

### Cell cytotoxicity

Cell viability following treatment of SW1353 cells with AML16 was measured by the MTT reduction assay. For this experiment, cells were seeded at 1 × 10^5^ cells/well in 96-well plates and cultured in DMEM. After treatment with ALM16 (25–1000 μg/ml) for 48 h, the medium was removed from the plate. The cells were washed twice with PBS and added with MTT solution (5 mg/ml in PBS) at 37 °C with 5% CO_2_ for 4 h. Cell viability was determined by measuring MTT formazan in each well wavelengths of 570 nm using microplate reader (Multiskan™, Thermo Scientific, CA, USA) and calculated relative to vehicle control cells.

### MMP-1, − 3 and − 13 levels in IL-1β treated SW1353 cells

To measure the levels of secreted MMP-1, − 3 and − 13 in IL-1β treated SW1353 cells, cells were seeded at 1 × 10^6^ cells/well in a 6-well plate. At confluence, cells were pretreated with sample for 30 min alone and further treated with IL-1β (20 ng/ml) for 24 h. The culture media were collected and the activity of MMPs were quantified using the SensoLyte® ELISA kit (AnaSpec, Fremont, CA, USA) following the manufacture’s instruction. The fluorescence of 5-FAM (fluorophore) was measured at excitation/emission absorbance of 490/520 nm using a fluorescence microplate reader (BioTek, Winooski, VT, USA).

### GAG contents in IL-1β treated SW1353 cells in alginate beads

The effects of ALM16 on proteoglycan degradation in SW1353 cells cultured in alginate beads were investigated. To produce extracellular matrix, SW1353 cells were cultured for 2 weeks in alginate beads, followed by the stimulation of IL-1β in the presence of ALM16 for 24 h. Beads were pretreated with various concentrations of ALM16 for 1 h before stimulation with IL-1β (20 ng/ml) for 24 h, and then, the amount of sulfated-GAG released in the cell suspension was quantified using a **Blyscan™ Glycosaminoglycan assay kit (Biocolor Ltd., UK)** according to the manufacturer’s instructions.

### Acetic acid induced writhing response in mice

Intraperitoneal injection (IP) of acetic acid causes abdominal contractions and induces a writhing response. The anti-analgesic activity of ALM16 was assessed by counting the contractions of the abdominal muscles, such as stretching, tension to one side, and extension of the hind legs, in response to an intraperitoneal injection of acetic acid (0.8%, 10 μl/g b.w.) in mice [24]. After a 1-week adaptation period, male ICR mice were randomly assigned to five groups (*n* = 8). After fating for 14 h, mice were orally administered A (400 mg/kg b.w.), L (400 mg/kg b.w.), ALM16 (100, 200 and 400 mg/kg b.w.) or celecoxib™ (100 mg/kg b.w.) 1 h prior to the acetic acid injection. All samples were dissolved in distilled water. The control group was received distilled water. The number of writhes was recorded 5 min after the acetic acid injection for 15 min.

### Carrageenan induced paw edema in mice

The anti-inflammatory activity of AML16 was evaluated by the carrageenan injection method [25]. After a 1-week adaptation period, male ICR mice were randomly assigned to five groups (*n* = 8). After 14 h of fating, mice were orally administered A (400 mg/kg b.w.), L (400 mg/kg b.w.), ALM16 (100, 200, and 400 mg/kg b.w.) or celecoxib™ (100 mg/kg b.w.). All samples were dissolved in distilled water. The control group was received distilled water. After 1 h of sample treatment, mice were subcutaneously injected with 1% carrageenan in 0.9% saline (25 μl/animal) into the left hind paw to induce acute phase inflammation. The thickness of the paw was measured 5 h after injection using a microcaliper. The change in paw thickness was calculated by subtracting the paw thickness before the injection from the paw thickness after the carrageenan injection at each time point.

### MIA-induced OA in rats and treatment

The MIA-induced OA rat model was used to investigate the protective effect of ALM16 on joint cartilage degradation. To induce OA, Wister rats were anesthetized with 1.2% avertin (2.5 ml/ 100 g b.w.) and received a single intra-articular injection of 1 mg/ 50 μl MIA (in PBS) into the right knee joint cavity using a Hamilton syringe (26 G) [26]. The control group was injected with an equivalent volume of saline. Seven days after induction of OA, MIA-injected rats were randomly divided into 8 groups (*n* = 8): treated with (1) normal, (2) control, (3) JOINS™ (as a positive control, 200 mg/kg b.w.), (4) A extract (400 mg/kg b.w.), (5) L extract (400 mg/kg b.w.) and (6–8) ALM16 (100, 200 and 400 mg/kg b.w., respectively). All samples (A, L, ALM16 and JOINS™) were dissolved in distilled water and were orally administered once daily for 14 days starting from 7 days after the MIA injection. The normal group was provided distilled water and did not receive the MIA injection. The weight of rats was measured twice per week for 2 weeks.

### Measurement of mechanical allodynia (von Frey test)

To measure the paw withdrawal threshold (PWT) for mechanical allodynia, the hind paw of rats was assessed using von Frey monofilaments (**Bioseb**®, Chaville, France). Briefly, rats were placed in a transparent plastic cage (20 × 12.5 × 20 cm) with a metal mesh bottom. Then, a von Frey monofilament was applied to the plantar mid surface of the hind foot for 3–4 s. The strength of the maximum filament used for von Frey testing was 15 g. Stimuli were applied at the same location with an interval of several seconds. The 50% PWT value of each group was measured using the up-down method of Dixon [27]. Positive responses included the abrupt withdrawal of the hind paw from the stimulus or a flinching behavior immediately following removal of the stimulus. The percent maximal possible effect (% MPE) of the testing compound was calculated according to the following formula: [(sample treated threshold) - (vehicle treated threshold)]/[(maximum threshold) - (vehicle treated threshold)] × 100%, where the maximum threshold was equal to 15 g.

### Histopathological analysis

Histological changes were analyzed to assess the effect of ALM16 on cartilage degeneration in the knee joint of MIA-induced OA rats. After 14 days of treatment, rats in all groups were anesthetized by exposure to ether (approximately 2–4%) presented on gauze inside a desiccator. The rats were removed from the desiccator and euthanized by cardiac bleeding. The tissue samples were then collected and subjected to a histological assessment of the affected medial condyle of each femur and tibia. Tissue was fixed with 10% formalin and decalcified. Tissue was then processed and embedded in paraffin wax. Tissue sections were then examined under a microscope after staining with hematoxylin and eosin (H&E). The histopathological changes of cartilage were quantitatively expressed simply by summing individual grades (score from 0 to 5) for subchondral bone changes, chondrocyte necrosis, cartilage erosion, osteophytes, and cartilage cleft, for a maximum score of 25 per section [28], where: 0 = normal; 1 = minimal, affecting the superficial zone only; 2 = mild invasion into the upper middle zone only; 3 = moderate invasion well into the middle zone; 4 = marked invasion into the deep zone but not to the tidemark; and 5 = severe full-thickness degradation to the tidemark. All of these histological evaluation procedures were performed by a board certified toxicologic pathologist in a blind manner.

### Statistical analysis

Semi-quantitative analysis was performed using non-parametric one-way analysis of variance following Tukey’s multiple comparison test (Graph-Pad Prism® Version 4.03). Data of all groups are presented as the mean values with the standard error of the mean (SEM). The difference between the data from the control and treated groups was assessed using non-paired Student’s *t*-test or the Mann-Whitney U test (Sigma-Stat version 10). *P*-values of *p < 0.05* were considered significant.

## Results

### HPLC-DAD quantification of AML16

**Fig. 1:**
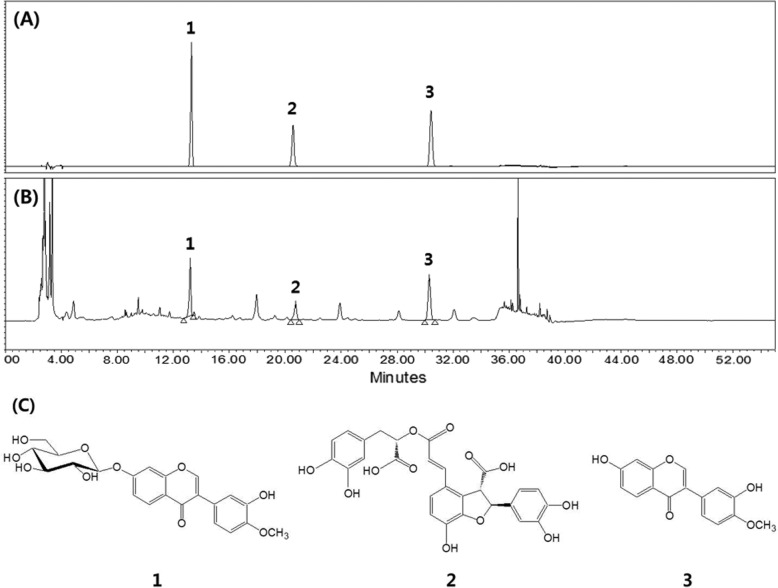
HPLC-DAD analysis of active compounds from ALM16. **a** HPLC chromatogram of (**a**) mixtures of authentic standard and (**b**) ALM16, analyzed on a YMC ODS-AM column. **c** The structures of calycosin-7-*O*-*β*-D-glucoside (1), lithospermic acid (2) and calycosin (3)

HPLC-based quantitative profiling of the active compounds from ALM16 was performed by comparing the retention time and UV spectra of standard compounds with those of ALM16 (Fig. 1). The contents of calycosin, calycosin-7-*O-β*-D-glucoside and lithospermic acid from ALM16 were measured to be 0.571, 0.809 and 0.168 mg/g, respectively (Fig. 1).

### Cell cytotoxicity of ALM16 on SW1353 cells

**Fig. 2:**
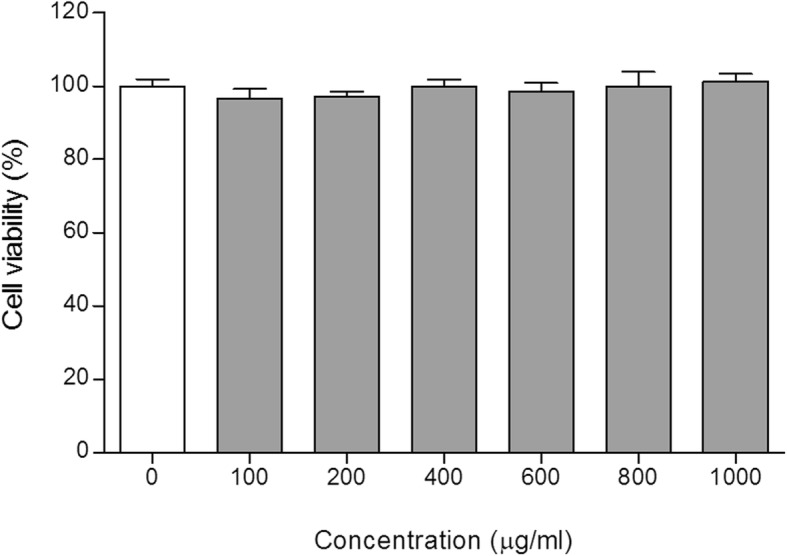
Cell cytotoxicity of ALM16 on SW1353 cells. Cells were seeded at 1 × 10^5^ cells/well in 96-well plates, and ALM16 (25–1000 μg/ml) was used to treat SW1353 cells for 48 h. Cell viability was investigated using the MTT assay. The results are expressed as the mean ± S.E.M of triple experiments

The cell cytotoxic effects of ALM16 on SW1353 cells after treating them with different concentrations of ALM16 were determined by the MTT reduction assay. As shown in Fig. 2, there was no significant difference on cell viability in all groups treated with ALM16 compared to vehicle control.

### Effects of ALM16 on the MMPs activities in IL-1β treated SW1353 cells

**Fig. 3:**
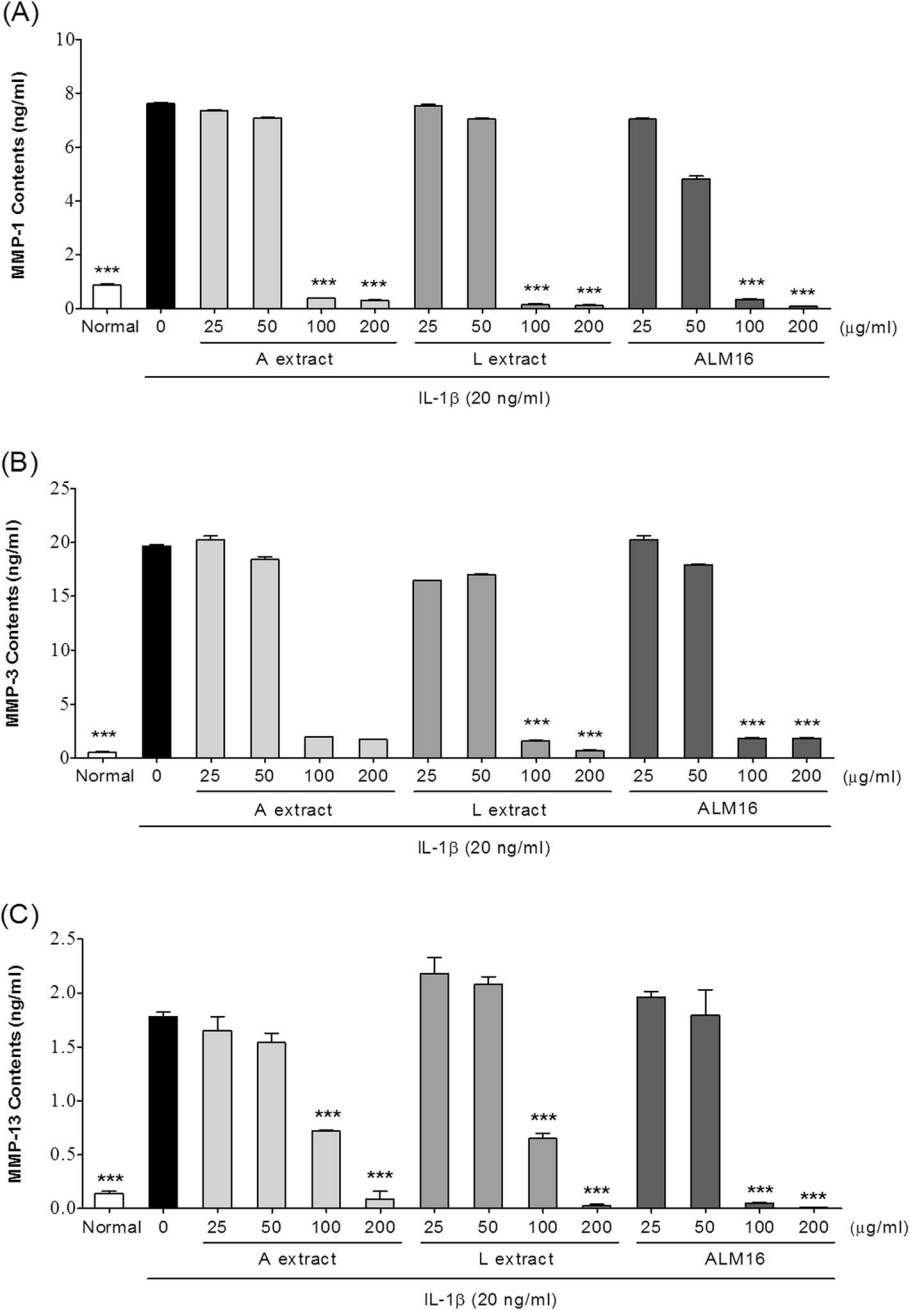
Effects of ALM16 on the activities of MMP-1, − 3 and − 13 in IL-1β-induced SW1353 cells. **a** MMP-1, (**b**) MMP-3 and (**c**) MMP-13 levels. Cells were pre-treated with A, L and ALM16 for 30 min and then further treated with IL-1β for 24 h. The content of MMPs in the cell supernatant was detected using a commercial ELISA kit method. The results are expressed as the mean ± S.E.M of triple experiments. ^*^p < 0.05; ^**^p < 0.01; ^***^p < 0.001 compared with IL-1β-treated control cells

To investigate the inhibitory effects of ALM16 against the increase of catabolic cartilage enzyme activities by IL-1β induction, the MMP concentrations in the culture supernatants of IL-1β (20 ng/ml)-induced SW1353 cells were calculated and are presented as ng total MMP_S_/1 × 10^6^ cells to standardize amounts between cultures. As shown in Fig. 3, treating cells with IL-1β remarkably increased (*p* < 0.001) the activation of MMPs (− 1, − 3 and − 13) compared with untreated cells. However, all of samples tested reduced these MMP activities in a dose-dependent manner. In particular, at concentrations above 100 μg/ml, ALM16 treatment dramatically reduced MMP activation, with an approximately 95% decrease (*p* < 0.001) compared with IL-1β-treated control cells. The IC_50_ values of ALM16 on MMP-1, − 3 and − 13 production were 54.36 ± 4.81, 69.69 ± 4.33 and 74.22 ± 9.90 ng/ml, respectively.

### Effects of ALM16 on GAG release in IL-1β treated SW1353 bead cells

**Fig. 4:**
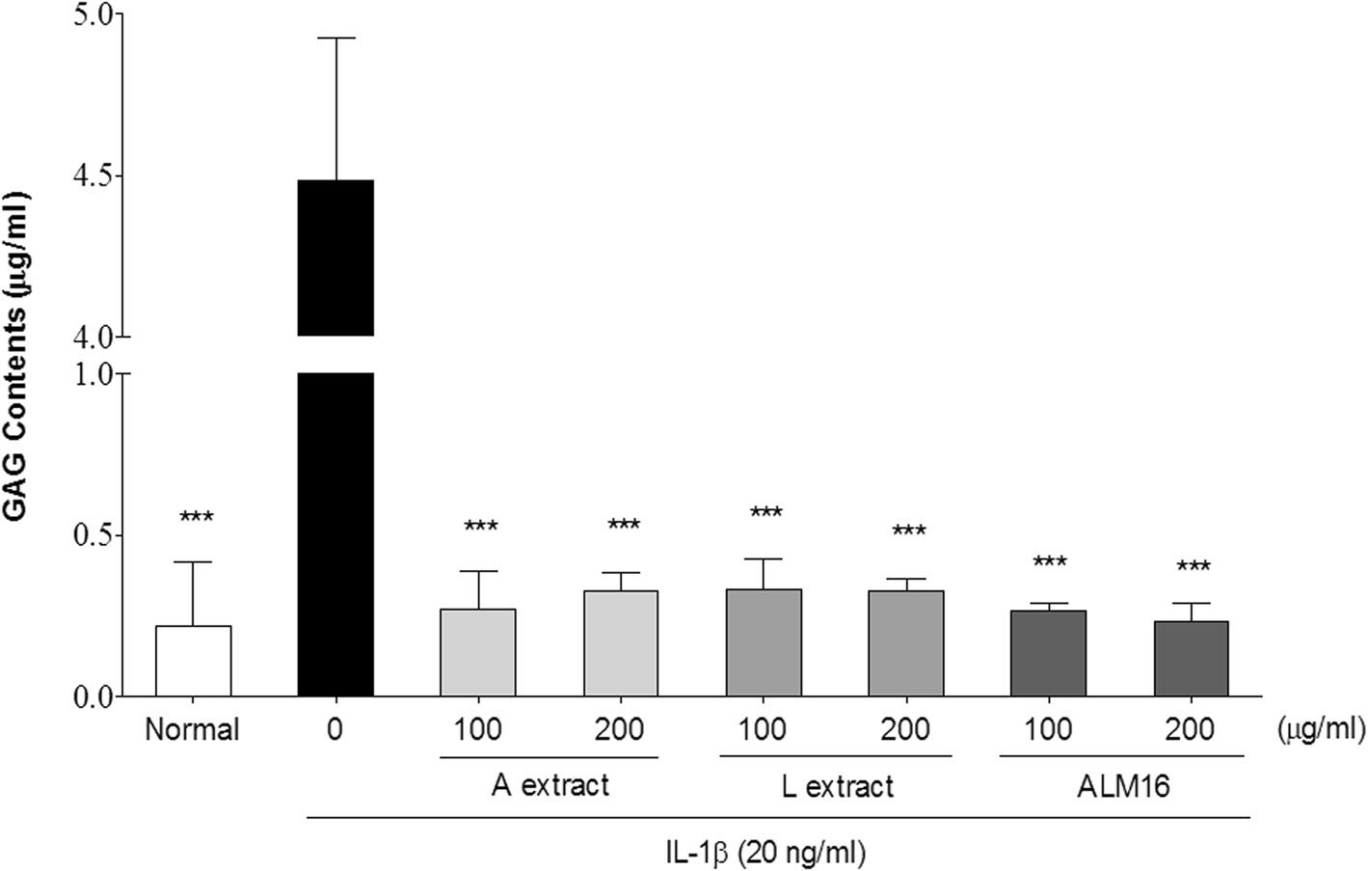
Effects of ALM16 on the GAG content in IL-1β-induced SW1353 alginate bead cells. Cells were pre-treated with A, L and ALM16 for 30 min and then further treated with IL-1β for 48 h. The GAG content in the cell supernatant was detected using a **GAG assay kit**. The results are expressed as the mean ± S.E.M of triple experiments. ^***^p < 0.001 compared with IL-1β-treated control cells

We investigated whether ALM16 could inhibit GAG degradation via IL-1β induction. Figure 4 shows that IL-1β (20 ng/ml) stimulation significantly increased GAG degradation (*p* < 0.001) from the cell supernatant of alginate beads compared to the unstimulated control. However, the degree of GAG degradation was significantly decreased in all sample treatment (*p* < 0.001). In the group treated with only IL-1β (4.48 ± 1.44 μg), the content of released GAGs was significantly greater than that of normal control (0.22 ± 0.19 μg). On the other hand, cells treated with A, L and ALM16 strongly inhibited the decomposition of GAG. The GAG levels obtained at the concentrations of ALM16 100 and 200 μg/ml of ALM16 were 0.26 ± 0.02 and 0.23 ± 0.05 μg, respectively.

### Anti-analgesic effect of ALM16 on the acetic acid-induced writhing response in mice

**Fig. 5:**
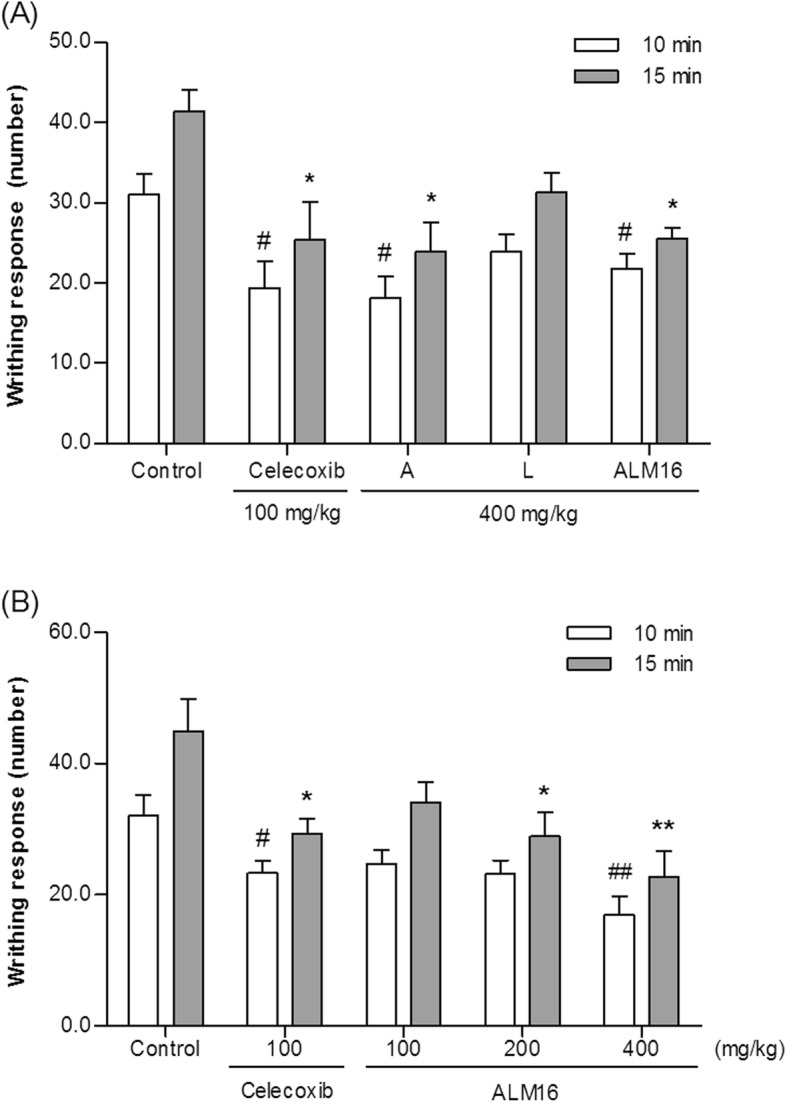
Analgesic effects of ALM16 in acetic acid-induced writhing mice. Writhing number was measured per 10 and 15 min after a 0.8% acetic acid injection. **a** Injection of A, L and ALM16 (400 mg/kg b.w.) or celecoxib (100 mg/kg b.w.). **b** Injection of ALM16 only in a dose dependent manner. The results are expressed as the mean ± S.E.M. (*n* = 8). Data were analyzed by one-way ANOVA Tukey’s test to compare all of the tested groups. ^#^*p* < 0.05, ^##^*p* < 0.01 and ^*^p < 0.05, ^**^p < 0.01 compared with the control group at 10 and 15 min after acetic acid injection, respectively

The analgesic effects of ALM16 were investigated according to the writhing response in acetic acid-induced mice. As shown in Fig. 5a, administration of 400 mg/kg A extract and ALM16 reduced the writhing response (*p* < 0.05), which was caused by abdominal contractions for 10 and 15 min, respectively. In Fig. 5b, ALM16 showed 23.9, 35.7% (*p* < 0.05) and 49.4% (*p* < 0.01) inhibition of writhing at doses of 100, 200 and 400 mg/kg b.w., respectively, as a percentage of the control 15 min after acetic acid injection. Celecoxib™ (100 mg/kg, b.w.), as a positive control, decreased writhing response by 27.2% (*p* < 0.05) and 34.7% (*p* < 0.05) for 10 and 15 min, respectively (Fig. 5b). This result suggests that ALM16 has a strong antinociceptive effect on the acetic acid-induced writhing response.

### Anti-inflammatory effect of AML16 on carrageenan-induced paw edema in mice

**Fig. 6:**
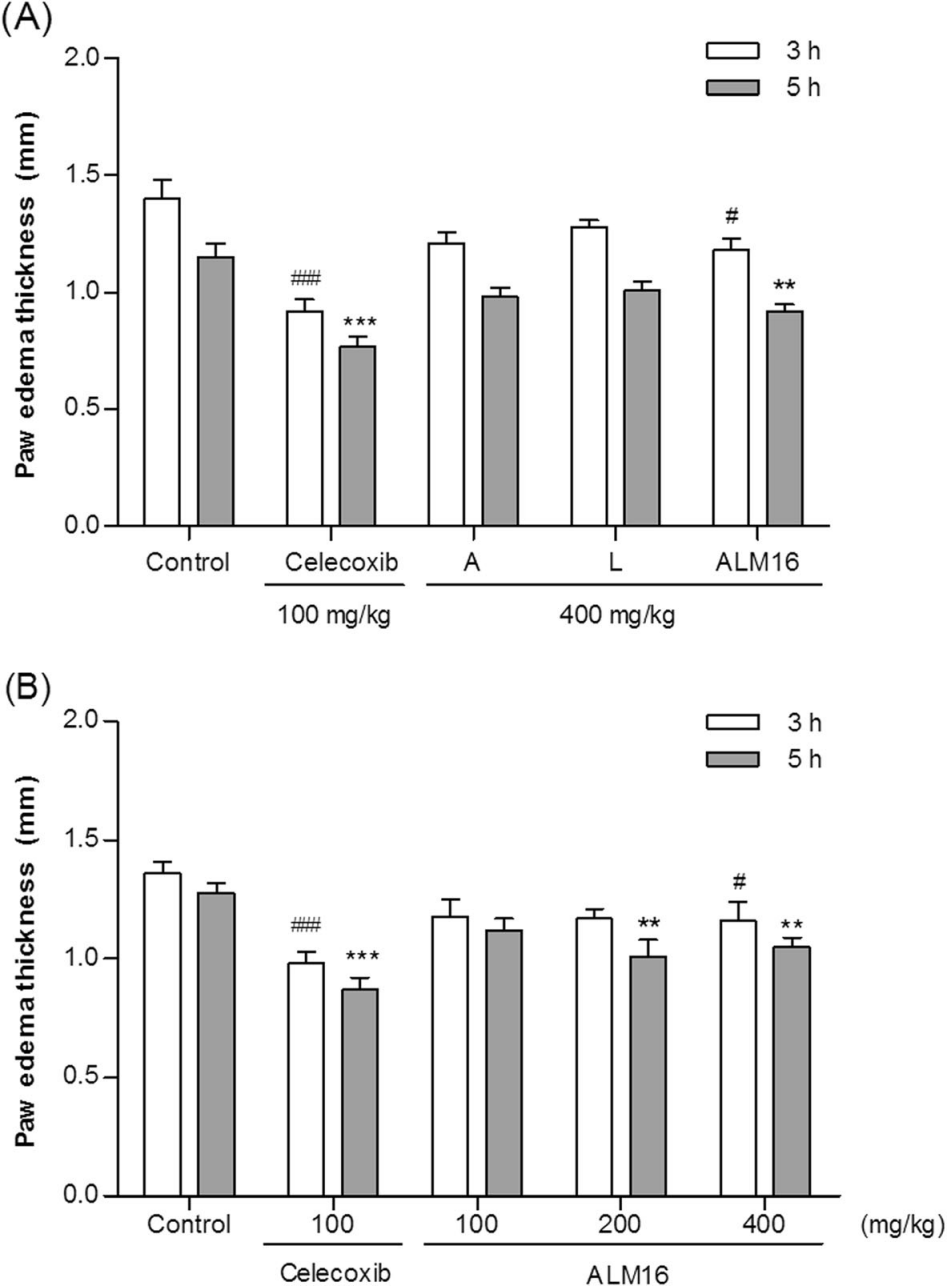
Anti-edematous effect of ALM16 on carrageenan-induced paw edema in mice. Edema thickness was measured 1 h after the 1% carrageenan injection. **a** Injection of A, L and ALM16 (400 mg/kg b.w.) or celecoxib (100 mg/kg b.w.). **b** Injection of ALM16 in a dose dependent manner. The results are expressed as the mean ± S.E.M (n = 8). Data were analyzed by one-way ANOVA Tukey’s test to compare all of the tested groups. ^#^*p* < 0.05, ^##^*p* < 0.01, ^###^*p* < 0.01 and ^*^p < 0.05, ^**^p < 0.01, ^***^p < 0.001 compared with the control group at 3 and 5 h after 1% carrageenan injection, respectively

Following treatment with each extract, ALM16 and celecoxib™, the thickness change of the paw in mice after carrageenan injection is shown in Fig. 6. In Fig. 6a, ALM16 led to a 20.6% (*p* < 0.01) reduction of paw thickness compared to the control 5 h after injection, which was time dependent. Celecoxib™ (100 mg/kg, b.w.), as a positive control, reduced paw thickness by 33.1% 5 h after injection. However, the A and L extracts had inhibitory effects of 15.2 and 12.1% 5 h after injection, respectively. Thus, administration of ALM16 was more effective than that of each individual extracts (A or L) treatment. In Fig. 6b, the rate of reduction of the thickness of the paw edema as a percentage of the control was 12.5, 19.5% (*p < 0.01*), and 20.3% (*p < 0.01*) for 100, 200 and 400 mg/kg b.w. ALM16, respectively, with dose dependence. From these results, the anti-inflammatory activity of ALM16 is shown to be caused by the synergistic effect of each extract.

### Change of mechanical allodynia in MIA-induced OA rats

**Fig. 7:**
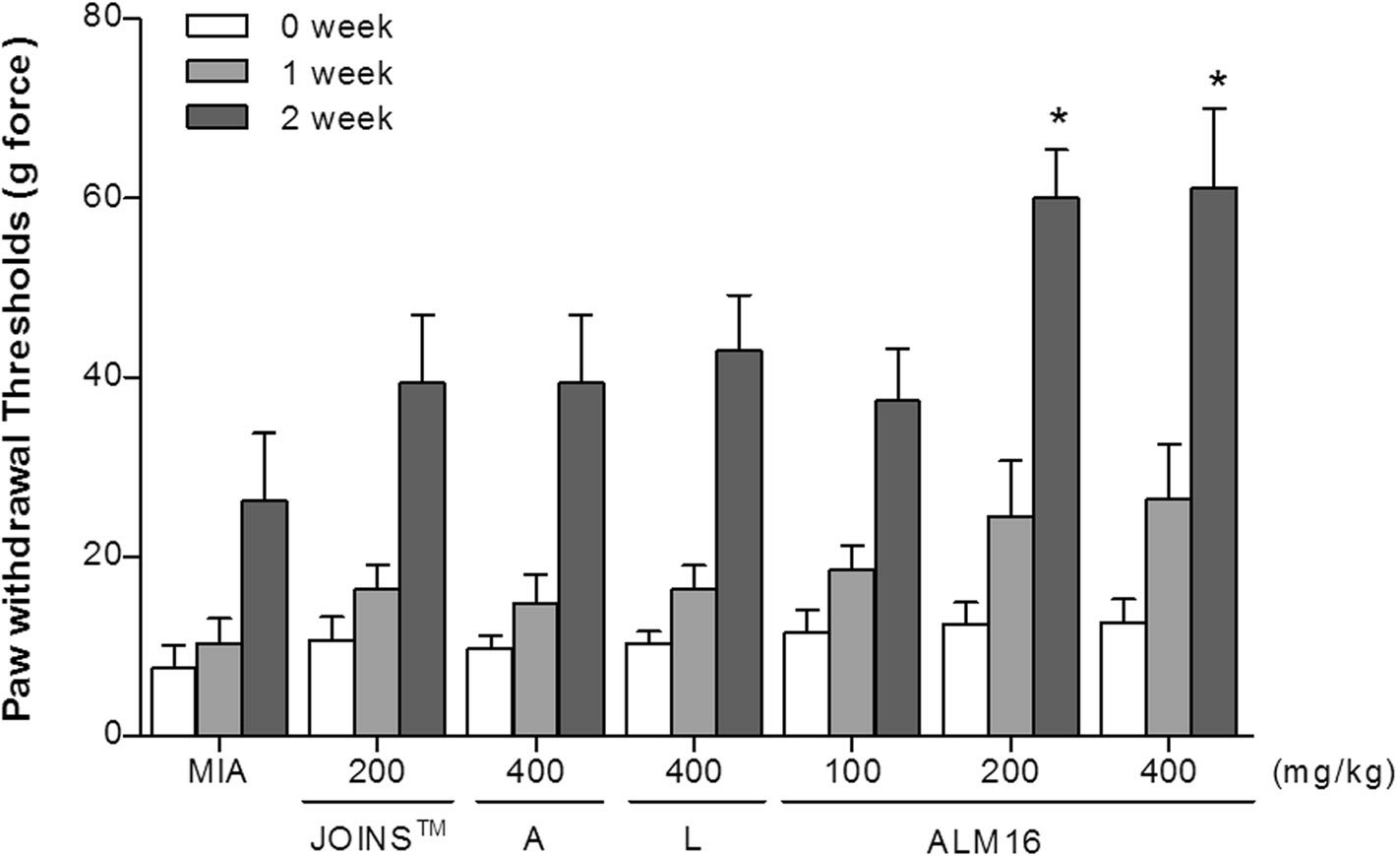
Effects of ALM16 on the changes of paw withdrawal thresholds in MIA-induced OA rats. The mechanical stimulus threshold of the hind paw was measured by von Frey filaments after injection of MIA in rats. The results are expressed as the mean ± S.E.M (*n* = 6). Data were analyzed by one-way ANOVA Tukey’s test to compare all of the tested groups. ^*^p < 0.05, ^**^p < 0.01, ^***^p < 0.001 compared with the MIA-injected control group

The mechanical threshold was measured using calibrated von Frey monofilaments, and the PWT was determined by increasing and decreasing the stimulus intensity and was estimated using Dixon’s up-down method. Changes in the PWT value of the hind paw in MIA-induced OA rats on days 0, 7 and 14 are shown in Fig. 7. The PWT was lower in the MIA-treated group than in the normal group, which had received a D.W. solution injection instead of MIA. The decrease in the PWT value of the control group (MIA-injected and D.W. solution-injected rats) continued throughout the trial period day 28. Treatment of MIA-injected rats with ALM16 dose-dependently increased the PWT value compared to the control groups at days 7 and 14. In addition, the ALM16 groups demonstrated a maximum effect (*p* < 0.05) of the PWT value compared with JOINS™ and each extract-treated group, and no significant difference was observed between doses of 200 and 400 mg/kg b.w. 14 days after the injection of MIA, the PWT value in the ALM16-treated groups was significantly higher than in the only MIA-treated group (*p* < 0.05). The ALM16 group had more potent analgesic effects than either extract (A or L) group. These results suggest that ALM16 has the ability to reduce mechanical allodynia and to exert synergistic actions.

### Histopathological pathogenesis in MIA-induced OA rats

**Fig. 8:**
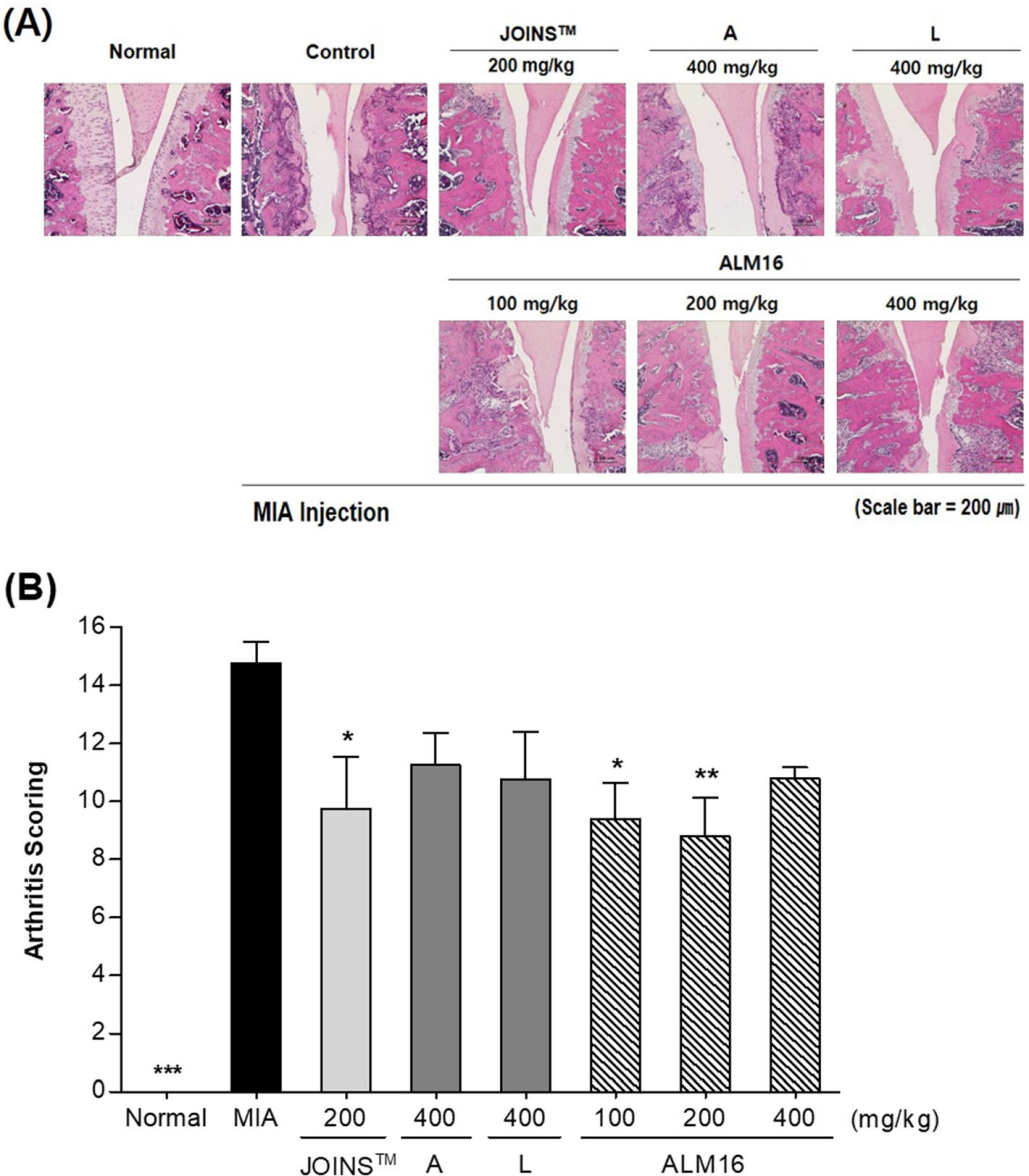
Histopathological changes of articular cartilage the femoral-tibial knee joint from MIA-induced OA rat. Wister rats were subjected to intra-articular injection of MIA (1 mg/animal) and fed orally with or without samples daily for 2 weeks. **a** Histological sections (× 200) were stained with hematoxylin & eosin (H&E) staining. **b** Histopathological changes are quantitatively expressed by arthritis scoring. The results are expressed as the mean ± S.E.M. Data were analyzed by one-way ANOVA Tukey’s test to compare all of the tested groups. ^*^p < 0.05, ^**^p < 0.01, ^***^p < 0.001 compared with the MIA-induced control group

The cartilage histology was evaluated in all groups of animals on day 21 in H&E-stained sections, and the respective microphotographs are shown in Fig. 8. The normal group did not show any remarkable lesions of arthritis histopathology. However, the MIA-injected control group had an irregular surface and extensive chondrocyte degeneration in the tibial plateau, collapse of necrotic articular cartilage, loss of chondrocyte cellular details, a focally extensive area of cartilage loss and degeneration in the femoral condyle. The subchondral bone in the tibial plateau showed increased osteoclastic activity, collapse and fragmentation with replacement by fibrous tissue. Compared to the arthritis score of control group (14.0 ± 2.5), each individual extract (A and L) and ALM16 treated groups (400 mg/kg) showed the low score on arthritis grade with no significant difference (11.3 ± 2.2, 10.8 ± 3.3 and 10.8 ± 0.8, *p* > 0.05). However, the ALM16 groups (100 and 200 mg/kg) had significantly lower scores (9.4 ± 2.8, *p* < 0.05 and 8.8 ± 3.0, *p* < 0.01) for the arthritis grade, which was lower than the arthritis score of JOINS™ 200 mg/kg group (9.8 ± 3.6, *p* < 0.05). These results showed that administration of 200 mg/kg ALM16 for a period of 2 weeks is demonstrably efficient at relieving histopathological changes in the MIA-induced OA rat.

## Discussion

OA is a degenerative chronic joint disease that causes joint pain, functional disabilities and progressive loss of articular cartilage induced by diverse factors [1]. This study demonstrated these properties using IL-1β-induced chondrocytes and an MIA-induced OA animal model, and the results showed that ALM16 significantly reduced pain, paw edema and OA pathological changes. Previous studies have reported health benefits of *A. membranaceus*, as a single extract or herbal mixtures, in various biological activities such as osteoprotective and anti-allergic rhinitis effects [29, 30]. In addition, our previous study showed that shikonin and acetylshikonin isolated from *L. erythrorhizon* had chondroprotective effects on MIA-induced OA rats [22]. Pseudoshikonin I isolated as a new compound from *L. erythrorhizon* showed also the inhibitory effect on MMPs [31]. However, none of the synergistic chondroprotective effects of the combination of *A. membranaceus* and *L. erythrorhizon* on OA have been studied. Therefore, in the present study, we examined the chondroprotective activity of a mixture of these medicinal herbal extracts and whether it had a synergistic effect on OA in vitro and in vivo.

Pro-inflammatory cytokines, such as IL-1β, are known to mediate the degradation of matrix proteins, such as proteoglycans and collagen, by activating of MMPs and apoptosis in chondrocytes. Previous studies have reported that the activities and/or gene expression of MMPs are key factors in the pathogenesis of OA. Accordingly, a number of studies have focused on identifying natural compounds that have potential inhibitory effects on gene expression or/and the catalytic activity of MMPs [9, 32]. It is also known that MMP-1, − 3 and MMP-13 are highly expressed in the articular cartilage of human OA patients. MMP is an enzyme involved in the reconstruction of the articular cartilage during growth, but in the case of arthritis, it is involved in the destruction of cartilage by inflammation responses. MMP-1 is the largest member of the MMP family and is synthesized by chondrocytes or fibroblasts in connective tissue. It is mainly degrading type II collagen in cartilage. MMP-3 (stromelysin-1) is secreted from chondrocytes and synovial cells and can cleaves a variety of matrix components by activating proMMP-1, resulting in fibrillation, erosion, cracking of cartilage tissue. MMP-13 is known as collagenase-3 and is increased during the process of early-onset OA. It has been reported that an MMP-13 target inhibitor efficiently blocks the matrix degradation in human OA cartilage, which is considered an effective target for OA [33]. IL-1β is known to upregulate the gene expression level of MMPs in chondrocytes and facilitates the progression of OA [34]. Gebauer et al. [35] reported that after IL-1β treatment, the human chondroma cell line SW1353 and human chondrocytes showed similar gene expression profiles, particularly for MMP-1, − 3 and − 13, which were strongly induced. In our previous study, we selected the effective ratio (7:3, ALM16) of combinations of two extracts using screening methods to measure the MMP-1, − 3 and − 13 activities [36]. ALM16 significantly and dose-dependently inhibited the increase in MMP activities caused by the IL-1β treatment in chondrocytes (Fig. 3). Interestingly, treatment of chondrocytes with ALM16 (100 and 200 μg/ml) significantly decreased the MMP-13 activity more than each extract individually. This result suggests that the chondroprotective effect of ALM16 may be due to inhibition of activities of MMP-1, − 3 and − 13.

The inhibitory activity of ALM16 on MMPs can be attributed to the individual or combined activities of the active compounds in ALM16. In this study, calycosin and calycosin-7-O-β-D-glucopyranoside from *A. membranaceus* were analyzed as the major active compounds in ALM16 based on the previous studies on anti-inflammatory and anti-arthritis effects [15, 16, 37]. Lithospermic acid was also evaluated as a major active compound of *L. erythrorhizon* in ALM16 on the basis of the reports on anti-inflammatory and MMP inhibitory effects [38, 39].

To determine where ALM16 protect structural breakdown of cartilage cell and matrix by MMPs reducing activity, the level of GAG dissolved from culture supernatant in chondrocyte alginate beads stimulated by IL-1β was quantified and compared. The concentration of each sample treatment is above 100 μg/ml, indicating the effect of inhibition of MMP activities. A, L and ALM16 treatment displayed strong GAG degradation inhibitory activity similar to those of normal group without IL-1β stimulation, which was the same result pattern as MMPs suppression activity. It is well recognized that IL-1β leads to cartilage damage via a large cascade of events [40]. Previous studies reported that treating chondrocytes with IL-1β increase GAGs degradation and release via increase MMPs activation [41, 42]. Therefore, these results clearly suggested that ALM16 has the ability to prevent the GAGs degradation as caused by IL-1β stimulation via inhibition of activities of MMP-1, − 3 and − 13.

Several analgesic drugs, including nonsteroidal anti-inflammatory drugs (NSAIDs), are commonly available for the treatment of pain and inflammation, but prolonged use of these drugs is associated with side effects such as gastric ulceration, nausea, and vomiting [34]. To discover new analgesic and osteoarthritis agents, extensive research is required to identify effective natural substances that do not have side effects. Thus, we evaluated the analgesic activity of ALM16 using an acetic acid-induced writhing model. It is well known that acetic acid induces abdominal contractions or writhing in mice because it leads to increased levels of pain mediators, such as PGE_2_. The acetic acid writhing model has been used to study the peripheral analgesic effects of drugs or as a screening model for assessing anti-inflammatory agents. The injection of an acetic acid solution into the abdominal cavity induces the release of endogenous mediators that stimulate the nociceptive neurons and indirectly acts as a cause for pain [43]. In our study, it was observed that when acetic acid-induced writhing mice were orally administered ALM16, the number of writhes was time-dependently decreased, and this effect was similar to that of celecoxib™ administration. Celecoxib™, which is a drug that relieves pain and inflammation by inhibiting MMP and nitric oxide (NO) production and improving joint function, is a selective NSAID used for symptomatic management of OA patients [44]. Additionally, the anti-inflammatory activity of ALM16 was further evaluated by its ability to inhibit paw edema induced by a carrageenan injection in mice. The carrageenan-induced paw edema model is widely accepted for use in evaluating anti-inflammatory effects. When the carrageenan is injected into the paw, muscles and joints, it causes early acute inflammation and converts to chronic inflammation after about 2 weeks [45, 46]. In this study, ALM16 administration markedly reduced the thickness of the paw edema induced by carrageenan after 3 and 5 h. Interestingly, ALM16 was more effective than administration of each extract alone. Therefore, in parallel with these findings, our results demonstrate that ALM16 attenuates pain symptoms and paw edema, thereby improving the articular cartilage in the local joints in OA animals. Recently, many studies have reported that the similar extracts from natural products can exert enhanced analgesic and anti-inflammatory activities [47, 48]. The mechanism underlying the synergistic effect is unknown, and further studies into the mechanism of cross-reaction or cross-acting of ALM16 are required. However, the synergistic or additive effect of ALM16 might be due to the combination ratio and concentration of the two extracts.

Recent studies have reported that natural compounds that have inhibitory effect on MMP expression and/or activities in chondrocytes can exert chondroprotective effects and potentially reduce joint pain in MIA-injected rats [49, 50]. An intra-articular injection of MIA, an inhibitor of glyceraldehyde-3-phosphate dehydrogenase (GAPDH) that induces dysfunction of chondrocytes, such as disruption of glycolysis and loss of chondrocytes, in articular cartilage produces symptoms of OA that are caused by progression of cartilage degeneration [51]. Therefore, the MIA-induced OA rat model is commonly used to study anti-osteoarthritis effects in preclinical studies. In this study, a 1 mg injection MIA was used identifying the OA degree based on our preliminary studies. MIA-injected OA rats were measured for body weight, the PWT value, and histopathological changes after oral administration of ALM16. Changes in body weight were monitored during the oral administration of all samples. None of the treated groups had significant differences in body weight compared with the control group during the experimental period (Additional file 1: Figure S1). This result indicates that oral administration of ALM16 does not have a toxic effect. Additionally, the analgesic effects of ALM16 against secondary mechanical hypersensitivity in OA were determined by the von Frey test. Our results demonstrated that administration of ALM16 at 200 and 400 mg/kg b.w. in the MIA-induced OA pain model significantly increased the reduction of the PWT values and was more effective at increasing the PWT values than the other treatment groups (A, L and JOINS™ groups). Several studies have reported that JOINS™, a drug combining the extracts of oriental herbs that are commonly and traditionally used for the treatment of OA patients in Korea, has cartilage protective effects by inhibiting ECM component degradation via inhibiting MMP expression and activity [52]. Therefore, these results support the evidence that ALM16 has a potential cartilage protective effect via analgesic actions in OA patients.

The MIA-injected OA model is known to have degenerative changes in cartilage containing subchondral bone changes, chondrocyte necrosis, cartilage erosion, osteophytes and cartilage clefts [28]. In this study, histopathological examination showed that MIA induced remarkable lesions in OA, including chondrocyte degeneration, collapse and fragmentation of subchondral bone, loss of chondrocytes, a focally extensive area of cartilage loss and degeneration in the femoral condyle. However, oral administration of ALM16 significantly mitigated MIA-induced histopathological lesions in cartilage. In particular, administration of ALM16 at a dose of 200 mg/kg led to a significantly lower histopathological score (for grade 5 major lesions) than the JOINS™ treated group. These results suggest that ALM16 has a potent protective effect on articular cartilage and that a dose of 200 mg/kg is effective in MIA-induced OA rats.

## Conclusions

Taken collectively, the results of the present studies suggest that application of ALM16 was not only be able to inhibit IL-1β induced increasing of MMPs activities in chondrocytes, but also mitigate as well as symptoms of OA including spontaneous pain, mechanical allodynia, paw edema and histopathological changes in established in vivo models. Although, whether it is necessary to further study on exact mechanism and on equally effects in clinical studies on anti-osteoarthritis effect of ALM16 against OA, based on these results, the ALM16 could be a potent candidate material for development of functional food that prevent or/and improve OA.

## Supplementary information

**Additional file 1: Figure S1.** Effects of ALM16 on the change of body weight in MIA-induced OA rats. Body weight was measured twice a week for 24 days. The results are expressed as the mean ± S.E.M (*n* = 6).

## Data Availability

The datasets used and/or analyzed during the current study are available from the corresponding author on reasonable request.

## Abbreviations

ECM: extracellular matrix
ELISA: enzyme-linked immunosorbent assay
GAG: Glycosaminoglycan
GAPDH: Glyceraldehyde-3-phosphate dehydrogenase
H&E: Hematoxylin and eosin
IL-1β: Interleukin-1β
IP: Intraperitoneal injection
MIA: Monosodium iodoacetate
MMPs: Matrix metalloproteinases
MTT: 3-[4,5-dimethylthiazol-2-yl]-2,5-diphenyltetrazolium bromide
NSAIDs: Nonsteroidal anti-inflammatory drugs
OA: Osteoarthritis
PWT: Paw withdrawal threshold

## References

[1] Aigner T, McKenna L (2002). Molecular pathology and pathobiology of osteoarthritic cartilage. Cell Mol Life Sci.

[2] Park JS, Lee HJ, Lee DY, Jo HS, Jeong JH, Kim DH, Nam DC, Lee CJ, Hwang SC (2015). Chondroprotective effects of wogonin in experimental models of osteoarthritis *in vitro* and *in vivo*. Biomol Ther.

[3] Mandelbaum B, Waddell D (2005). Etiology and pathophysiology of osteoarthritis. Orthopedics.

[4] Braza-Boils A, Ferrandiz ML, Terencio MC, Alcaraz MJ (2012). Analysis of early biochemical markers and regulation by tin protoporphyrin IX in a model of spontaneous osteoarthritis. Exp Gerontol.

[5] Tanaka S, Hamanishi C, Kikuchi H, Fukuda K (1998). Factors related to degradation of articular cartilage in osteoarthritis: a review. Semin Arthritis Rheum.

[6] Wu YS, Hu YY, Yang RF, Wang Z, Wei YY (2007). The matrix metalloproteinases as pharmacological target in osteoarthritis: Statins may be of therapeutic benefit. Med Hypotheses.

[7] Nagase H, Woessner JF (1999). Matrix metalloproteinases. J Biol Chem.

[8] Murphy G, Knäuper V, Atkinson S, Butler G, English W, Hutton M, Stracke J, Clark I (2002). Matrix metalloproteinases in arthritic disease. Arthritis Res.

[9] Li NG, Shi ZH, Tang YP, Wang ZJ, Song SL, Qian LH, Qian DW, Duan JA (2011). New hope for the treatment of osteoarthritis through selective inhibition of MMP-13. Curr Med Chem.

[10] Fu J, Wang Z, Huang L, Zheng S, Wang D, Chen S, Zhang H, Yang S (2014). Review of the botanical characteristics, phytochemistry, and pharmacology of *Astragalus membranaceus* (Huangqi). Phytother Res.

[11] Bedir E, Pugh N, Calis I, Pasco DS, Khan IA (2000). Immunostimulatory effects of cycloartane-type triterpene glycosides from *astragalus* species. Biol Pharm Bull.

[12] Chan JY, Lam FC, Leung PC, Che CT, Fung KP (2009). Antihyperglycemic and antioxidative effects of an herbal formulation of *Radix Astragali, Radix Codonopsis* and *Cortex Lycii* in a mouse model of type 2 diabetes mellitus. Phytother Res.

[13] Shi R, He L, Hu Y, Yi N, Weng S, Cao Y (2001). The regulatory action of *Radix Astragali* on M-cholinergic receptor of the brain of senile rats. J Tradit Chin Med.

[14] Cho WC, Leung KN (2007). *In vitro* and *in vivo* anti-tumor effects of *Astragalus membranaceus*. Cancer Lett.

[15] Nigorikawa K, Yoshikawa K, Sasaki T, Iida E, Tsukamoto M, Murakami H, Maehama T, Hazeki K, Hazeki O (2006). A naphthoquinone derivative, shikonin, has insulin-like actions by inhibiting both phosphatase and tensin homolog deleted on chromosome 10 and tyrosine phosphatases K. Mol Pharmacol.

[16] Choi YH, Kim GS, Choi JH, Jin SW, Kim HG, Han Y, Lee DY, Choi SI, Kim SY, Ahn YS, Lee KY, Jeong HG (2016). Ethanol extract of *Lithospermum erythrorhizon* Sieb. et Zucc. promotes osteoblastogenesis through the regulation of Runx2 and Osterix. Int J Mol Med.

[17] Han J, Weng X, Bi K (2008). Antioxidants from a Chinese medicinal herb-*Lithospermum erythrorhizon*. Food Chem.

[18] Staniforth V, Wang SY, Shyur LF, Yang NS (2004). Shikonins, phytocompounds from *Lithospermum erythrorhizon*, inhibit the transcriptional activation of human tumor necrosis factor α promoter *in vivo*. J Biol Chem.

[19] Andújar I, Recio MC, Giner RM, Ríos JL (2013). Traditional chinese medicine remedy to jury: the pharmacological basis for the use of shikonin as an anticancer therapy. Curr Med Chem.

[20] Choi SI, Park SR, Heo TR (2005). Inhibitory effect of *Astragali* radix on matrix degradation in human articular cartilage. J Microbiol Biotechnol.

[21] Choi SI, Heo TR, Min BH, Cui JH, Choi BH, Park SR (2007). Alleviation of osteoarthritis by calycosin-7-*O*-beta-D-glucopyranoside (CG) isolated from *Astragali* radix (AR) in rabbit osteoarthritis (OA) model. Osteoarthr Cartil.

[22] Kim GS, Kim HJ, Lee DY, Choi SM, Lee SE, Noh HJ, Choi JG, Choi SI (2013). Effects of supercritical fluid extract, shikonin and acetylshikonin from *Lithospermum erythrorhizon* on chondrocytes and MIA-induced osteoarthritis in rats. Korean J Medicinal Crop Sci.

[23] Mok SS, Masuda K, Hauselmann HJ, Aydelotte MB, Thonar EJ (1994). Aggrecan synthesized by mature bovine chondrocytes suspended in alginate; identification of two distinct metabolic matrix pools. J Biol Chem.

[24] Mishra D, Ghosh G, Kumar PS, Panda PK (2011). An experimental study of analgesic activity of selective COX-2 inhibitor with conventional NSAIDs. Asian J Pharm Clin Res.

[25] Obiri DD, Osafo N (2013). Aqueous ethanol extract of the fruit of *Xylopia aethiopica* (Annonaceae) exhibits anti-anaphylactic and anti-inflammatory actions in mice. J Ethnopharmacol.

[26] Bove SE, Calcaterra SL, Brooker RM, Huber CM, Guzman RE, Juneau PL, Schrier DJ, Kilgore KS (2003). Weight bearing as a measure of disease progression and efficacy of anti-inflammatory compounds in a model of monosodium iodoacetate-induced osteoarthritis. Osteoarthr Cartil.

[27] Chaplan SR, Bach FW, Pogrel JW, Chung JM, Yaksh TL (1994). Quantitative assessment of tactile allodynia in the rat paw. J Neurosci Methods.

[28] Guzman RE, Evans MG, Bove S, Morenko B, Kilgore K (2003). Mono-iodoacetate-induced histologic changes in subchondral bone and articular cartilage of rat femorotibial joints: an animal model of osteoarthritis. Toxicol Pathol.

[29] Huh JE, Kim SJ, Kang JW, Nam DW, Choi DY, Park DS, Lee JD (2015). The standardized BHH10 extract, a combination of *Astragalus membranaceus, Cinnamomum cassia,* and *Phellodendron amurense*, reverses bone mass and metabolism in a rat model of postmenopausal osteoporosis. Phytother Res.

[30] Matkovic Z, Zivkovic V, Korica M, Plavec D, Pecanic S, Tudoric N (2010). Efficacy and safety of *Astragalus membranaceus* in the treatment of patients with seasonal allergic rhinitis. Phytother Res.

[31] Lee DY, Choi SI, Han SH, Lee YJ, Choi JG, Lee YS, Choi JH, Lee SE, Kim GS (2016). Potential of Pseudoshikonin I isolated from Lithospermi Radix as inhibitors of MMPs in IL-1β-induced SW1353 cells. Int J Mol Sci.

[32] Chen JJ, Huang JF, Du WX, Tong PJ (2014). Expression and significance of MMP3 in synovium of knee joint at different stage in osteoarthritis patients. Asian Pac J Trop Med.

[33] Takahashi K, Goomer RS, Harwood F, Kubo T, Hirasawa Y, Amiel D (1999). The effects of hyaluronan on matrix metalloproteinase-3 (MMP-3), interleukin-1β (IL-1β), and tissue inhibitor of metalloproteinase-1 (TIMP-1) gene expression during the development of osteoarthritis. Osteoarthr Cartil.

[34] Wang M, Sampson ER, Jin H, Li J, Ke QH, Im HJ, Chen D (2013). MMP13 is a critical target gene during the progression of osteoarthritis. Arthritis Res Ther.

[35] Gebauer M, Saas J, Sohler F, Haag J, Soder S, Pieper M, Bartnik E, Beninga J, Zimmer R, Aigner T (2005). Comparison of the chondrosarcoma cell line SW1353 with primary human adult articular chondrocytes with regard to their gene expression profile and reactivity to IL-1β. Osteoarthr Cartil.

[36] Choi DJ, Choi BR, Lee DY, Choi SI, Lee YS, Kim GS (2019). Inhibitory Effect of Mixed Extracts Obtained from Astragali Radix and Lithospermi Radix on Matrix Metalloproteinases in IL-1β-induced SW1353 Cells and Quantitative Analysis of Active Compounds. Korean J Medicinal Crop Sci.

[37] Liu XY, Xu L, Wang Y, Li JX, Zhang Y, Zhang C, Wang SS, Zhang XM (2017). Protective effects of total flavonoids of *Astragalus* against adjuvant-induced arthritis in rats by regulating OPG/RANKL/NF-κB pathway. Int Immunopharmacol.

[38] Liu X, Chen R, Shang Y, Jiao B, Huang C (2008). Lithospermic acid as a novel xanthine oxidase inhibitor has anti-inflammatory and hypouricemic effects in rats. Chem Biol Interact.

[39] Yoo HG, Lee BH, Kim W, Lee JS, Kim GH, Chun OK, Koo SI, Kim DO (2014). *Lithospermum erythrorhizon* extract protects keratinocytes and fibroblasts against oxidative stress. J Med Food.

[40] Wood DD, Ihrie EJ, Dinarello CA, Cohen PL (1983). Isolation of an interleukin-1-like factor from human joint effusions. Arthritis Rheum.

[41] Moon MH, Jeong JK, Lee YJ, Seol JW, Park SY (2012). Sphingosine-1-phosphate inhibits interleukin-1β-induced inflammation in human articular chondrocytes. Int J Mol Med.

[42] Lee JH, Shehzad O, Ko SK, Kim YS, Kim HP (2015). Matrix metalloproteinase-13 downregulation and potential cartilage protective action of the Korean Red Ginseng preparation. J Ginseng Res.

[43] Bighetti EJ, Hiruma-Lima CA, Gracioso JS, Brito AR (1999). Anti-inflammatory and antinociceptive effects in rodents of the essential oil of *Croton cajucara* Benth. J Pharm Pharmacol.

[44] Gordo AC, Walker C, Armada B, Zhou D (2017). Efficacy of celecoxib versus ibuprofen for the treatment of patients with osteoarthritis of the knee: A randomized double-blind, non-inferiority trial. J Int Med Res.

[45] Jeong JH, Moon SJ, Jhun JY, Yang EJ, Cho ML, Min JK (2015). Eupatilin exerts antinociceptive and chondroprotective properties in a rat model of osteoarthritis by downregulating oxidative damage and catabolic activity in chondrocytes. PLoS One.

[46] Posadas I, Bucci M, Roviezzo F, Rossi A, Parente L, Sautebin L, Cirino G (2004). Carrageenan-induced mouse paw oedema is biphasic, age-weight dependent and displays differential nitric oxide cyclooxygenase-2 expression. Br J Pharmacol.

[47] Gupta AK, Parasar D, Sagar A, Choudhary V, Chopra BS, Garg R, Ashish, Khatri N (2015). Analgesic and anti-inflammatory properties of gelsolin in acetic acid induced writhing, tail immersion and carrageenan induced paw edema in mice. PLoS One.

[48] Mondal H, Saha S, Awang K, Hossain H, Ablat A, Islam MK, Jahan IA, Sadhu SK, Hossain MG, Shilpi JA, Uddin SJ (2014). Central-stimulating and analgesic activity of the ethanolic extract of *Alternanthera sessilis* in mice. BMC Complement Altern Med.

[49] Kim J, Yang S, Choi CY (2016). The Evaluation of the effect of herbal extract on osteoarthritis: *In vitro* and *in vivo* study. Prev Nutr Food Sci.

[50] Bahtiar A, Nurazizah M, Roselina T, Tambunan AP, Arsianti A (2017). Ethanolic extracts of babandotan leaves (*Ageratum Conyzoides* L.) prevents inflammation and proteoglycan degradation by inhibiting TNF-alpha and MMP-9 on osteoarthritis rats induced by monosodium iodoacetate. Asian Pac J Trop Med.

[51] Orita S, Ishikawa T, Miyagi M, Ochiai N, Inoue G, Eguchi Y, Kamoda H, Arai G, Toyone T, Aoki Y, Kubo T, Takahashi K, Ohtori S (2011). Pain-related sensory innervation in monosodium iodoacetate-induced osteoarthritis in rat knees that gradually develops neuronal injury in addition to inflammatory pain. BMC Musculoskelet Disord.

[52] Choi JH, Choi JH, Kim DY, Yoon JH, Youn HY, Yi JB, Rhee HI, Ryu KH, Jung K, Han CK, Kwak WJ, Cho YB (2002). Effects of SKI 306X, a new herbal agent, on proteoglycan degradation in cartilage explant culture and collagenase-induced rabbit osteoarthritis model. Osteoarthr Cartil.

