# Supplementary material for: Cartilage protective and anti-analgesic effects of ALM16 on monosodium iodoacetate induced osteoarthritis in rats
Source: BMC Complement Altern Med. 2019 Nov 21;19:325. doi: 10.1186/s12906-019-2746-7 (PMC6873692; doi:10.1186/s12906-019-2746-7)
Supplement: Supplementary file 1 — Additional file 1: Figure S1. Effects of ALM16 on the change of body weight in MIA-induced OA rats. Body weight was measured twice a week for 24 days. The results are expressed as the mean ± S.E.M (n = 6). [file 12906_2019_2746_MOESM1_ESM.docx]

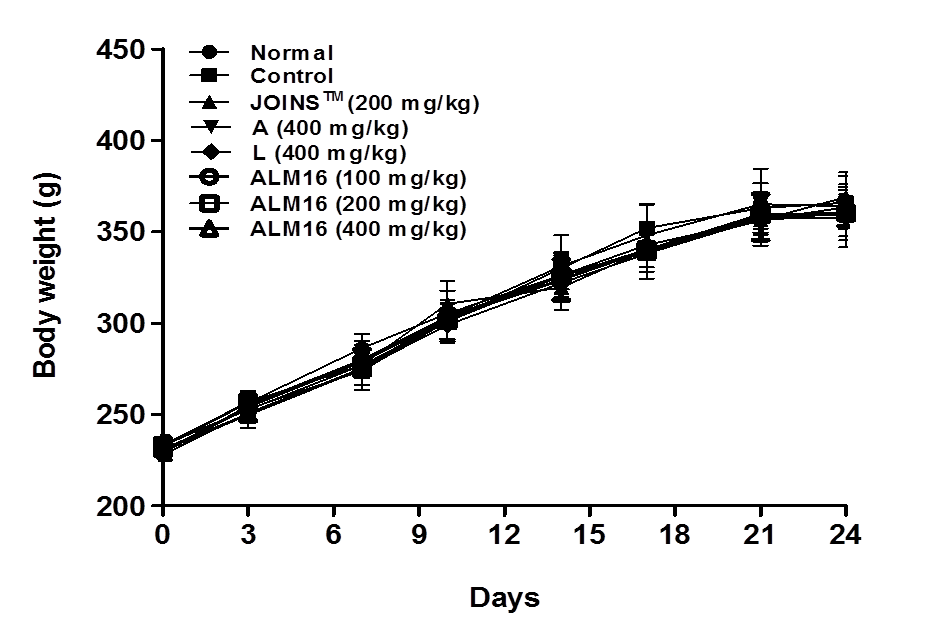


**Figure S1. Effects of ALM16 on the change of body weight in MIA-induced OA rats.** Body weight was measured twice a week for 24 days. The results are expressed as the mean ± S.E.M (n = 6).
